# Impact of MetS on Long-Term Prognosis Among STEMI Patients Treated with pPCI—Ten-Year Follow-Up Study

**DOI:** 10.3390/medsci14020268

**Published:** 2026-05-21

**Authors:** Milan B. Lović, Dragan B. Đorđević, Sandra B. Šarić, Ivan S. Tasić, Dejana D. Isaković, Jovana Lj. Kostić

**Affiliations:** 1 Institute for Prevention and Cardiovascular Rehabilitation, Srpskih Junaka 2, Niška Banja, 18205 Niš, Serbia; dragan.djordjevic@radonnb.co.rs (D.B.Đ.); sandra.saric@radonnb.co.rs (S.B.Š.); ivan.tasic@radonnb.co.rs (I.S.T.); dejana.isakovic@radonnb.co.rs (D.D.I.); jovana.kostic@radonnb.co.rs (J.L.K.); 2 Faculty of Medicine, University of Niš, Bulevar Dr Zorana Đinđića 81, 18000 Niš, Serbia

**Keywords:** metabolic syndrome, ST-segment elevation myocardial infarction, primary percutaneous coronary intervention, heart failure, major adverse cardiovascular events, obesity paradox, competing risk analysis, machine learning, SHAP, Random Survival Forest, long-term follow-up

## Abstract

Background/Objectives: Metabolic syndrome (MetS) affects more than 1.5 billion adults worldwide and is present in 37–70% of STEMI patients. Its ten-year prognostic value after primary PCI—particularly for heart failure, which is rarely examined as a primary endpoint—remains incompletely characterized. Methods: In total, 506 STEMI patients treated with primary PCI (December 2009–June 2010) were followed for ten years. MetS was defined at admission using AHA/NHLBI criteria. Co-primary endpoints were all-cause mortality, MACE, and hospitalization for heart failure. Multivariable Cox regression was adjusted for sex, age, LVEF, previous MI, Killip class, and multivessel disease. Four ML models were evaluated by 10-fold stratified cross-validation with SHAP-based feature, with a Fine–Gray subdistribution-hazard sensitivity analysis for heart failure. Feature attribution used TreeSHAP on XGBoost and permutation importance on a Random Survival Forest. Results: MetS(+) patients were older, more frequently female, and had higher SYNTAX scores (all *p* < 0.05). MetS was present in 216 patients (42.7%). It did not independently predict mortality (HR 1.09, *p* = 0.66) but did predict MACE (HR 1.47, *p* = 0.028) and heart failure hospitalization (cause-specific HR 2.86, 95% CI 1.57–5.22; Fine–Gray HR 2.61, 95% CI 1.44–4.75; both *p* ≤ 0.002). The null mortality finding coincided with differential statin discontinuation and a selective obesity paradox: in non-obese patients, MetS doubled mortality (42.9% vs. 21.1%, *p* = 0.008), while in obese patients, the effect disappeared (26.5% vs. 23.2%, *p* = 0.529). Two independent ML frameworks ranked the cumulative number of MetS criteria—rather than the binary diagnosis—among the leading individual-level features for heart failure prediction (Random Survival Forest c-index 0.843). Conclusions: In primary PCI-treated STEMI survivors, MetS independently predicts ten-year MACE and heart failure but not mortality. The number of MetS criteria at baseline, rather than the binary classification, was more strongly associated with heart failure risk; whether prospective modification of individual components reduces this risk requires dedicated interventional studies. The lean MetS-positive phenotype may represent a candidate subgroup warranting further investigation.

## 1. Introduction

Metabolic syndrome—characterized by central obesity, atherogenic dyslipidemia, hypertension, and impaired fasting glucose—has spread as a global epidemic, with recent estimates suggesting that more than 1.5 billion adults now meet diagnostic criteria [1]. In 2025, the European Atherosclerosis Society moved beyond viewing it as a risk factor cluster and reframed it as a staged systemic metabolic disorder, with heart failure and advanced coronary artery disease as the most severe downstream consequences [2]. The link with acute coronary disease is well established; between 37% and over 60% of patients presenting with acute coronary syndromes meet criteria for the syndrome [3,4], a co-prevalence consistent with the role of insulin resistance, endothelial dysfunction, and a prothrombotic milieu in driving plaque rupture [5].

However, the long-term prognostic role of metabolic syndrome after STEMI remains less clear. A recent meta-analysis of eleven studies confirmed an association with major adverse cardiovascular events (MACE) but found the evidence for long-term mortality inconsistent [6], and individual cohorts have produced divergent results [7,8]. Two methodological problems likely contribute to this picture. First, follow-up has typically been short, as most published studies report one- to three-year outcomes, with only a handful extending beyond five years; yet Dohi and colleagues showed that mortality curves for MetS and non-MetS patients begin to separate only after five years, suggesting that shorter studies systematically underestimate the long-term burden [9]. Second, the obesity paradox—the consistent observation that obese STEMI patients have lower long-term mortality than their normal weight counterparts [10]—is rarely accounted for in MetS analyses, despite the fact that most MetS patients in Western cohorts are overweight or obese.

Heart failure has received the least attention among post-STEMI endpoints in this literature, even though it has become increasingly relevant as primary PCI has improved early survival and patients live long enough for chronic complications to emerge. Metabolic syndrome is biologically plausible as a driver of post-infarction heart failure, as insulin resistance impairs myocardial glucose uptake, forcing a shift to fatty acid oxidation with mitochondrial dysfunction, and AGE–RAGE-mediated signaling promotes interstitial fibrosis [11,12]. Superimposed on the ischemic remodeling that follows STEMI, these mechanisms may produce a synergistic trajectory toward heart failure that has not been examined over a ten-year horizon.

We previously followed a prospective cohort of 531 STEMI patients treated with primary PCI for four years and reported that metabolic syndrome was independently associated with MACE (HR 1.83) but not with mortality [13]. The present study aimed to extend that follow-up to ten years in 506 patients with complete outcome data, using hospitalization for heart failure as the new primary endpoint. Three additional questions are addressed: whether MetS independently predicts ten-year all-cause mortality and MACE; whether obesity modifies the MetS–mortality relationship in the directions suggested by the obesity paradox literature; and whether the binary MetS diagnosis or the cumulative number of fulfilled criteria is the more informative individual-level prognostic signal, evaluated using an explainable machine learning approach (XGBoost with SHAP attribution and a Random Survival Forest sensitivity analysis).

## 2. Materials and Methods

### 2.1. Study Population

Between December 2009 and June 2010, 531 consecutive patients with ST-segment elevation myocardial infarction (STEMI) underwent primary percutaneous coronary intervention (pPCI) at the University Clinical Centre of Serbia in Belgrade and were enrolled in this prospective cohort. The four-year outcomes of this cohort have been reported previously [13]; the present analysis extends the follow-up to ten years.

Patients were eligible if they presented within 12 h of chest pain onset, had ST-segment elevation in at least two contiguous leads, and underwent successful pPCI of the infarct-related artery (TIMI 3 flow at the end of the procedure). All patients received aspirin, clopidogrel, and unfractionated heparin before the procedure and were managed according to the 2008 ESC STEMI guidelines [14]. Procedures were performed via the femoral route using a standardized technique, and a coronary stent was implanted in every patient. Patients were excluded if they had received thrombolysis prior to admission, had a TIMI flow < 3 at the end of the procedure, were younger than 18 years, and were unable to provide informed consent.

Of the 531 enrolled patients, 24 were lost to follow-up during the ten-year observation period (alive but unreachable by telephone or at their registered home addresses), and one additional patient was excluded because follow-up duration could not be determined. The final analytical sample comprised 506 patients (Figure S1, Supplementary Material, CONSORT flow diagram).

The protocol was approved by the Institutional Review Board of the Faculty of Medicine at the University of Belgrade (no. 29/XI-20, November 2013) and was conducted in accordance with the Declaration of Helsinki. All patients provided written informed consent after clinical stabilization.

### 2.2. Data Collection

Baseline data were collected prospectively by trained research staff who were blinded to the study aims and outcome status. Demographics, anthropometric measurements (height, weight, waist circumference, body mass index), cardiovascular risk factors, prior cardiovascular history, comorbidities, and clinical presentation data (Killip class, symptom-to-balloon time, infarct territory, culprit artery) were recorded using standardized instruments.

Serum creatine kinase (CK) and CK-MB were serially measured during the first 48 h after symptom onset, and peak values were recorded. Fasting glucose, HDL cholesterol, and triglycerides were measured from a fasting blood draw obtained on the morning of the final inpatient day. Left ventricular ejection fraction (LVEF) was determined by transthoracic echocardiography during the index admission, in accordance with ACC/AHA/ASE guidelines [15]. The SYNTAX score was calculated from the diagnostic coronary angiogram by two interventional cardiologists blinded to clinical data, with disagreements resolved by consensus.

### 2.3. Definition of Metabolic Syndrome

Metabolic syndrome (MetS) was defined by the AHA/NHLBI criteria [16], which require at least three of the following five components: abdominal obesity (waist circumference ≥ 102 cm in men, ≥88 cm in women); elevated triglycerides (≥1.7 mmol/L or lipid-lowering therapy); low HDL cholesterol (<1.03 mmol/L in men, <1.29 mmol/L in women, or therapy); arterial hypertension (≥130/85 mmHg or antihypertensive therapy); and elevated fasting glucose (≥5.6 mmol/L or antidiabetic therapy).

MetS status was assessed only at the index hospitalization, using fasting laboratory values from the final inpatient blood draw. Patients were classified as MetS-positive (n = 216, 42.7%) or MetS-negative (n = 290, 57.3%). The number of criteria met (range 0–5) was retained as a continuous variable for the dose–response and machine learning analyses.

### 2.4. Follow-Up and Outcome Definitions

Follow-up visits were scheduled at 1, 6, 12, 24, 36, 48, 60, 96, and 120 months after the index event. Each visit included a clinical examination, electrocardiography, and a review of current medications. Patients who missed scheduled visits were contacted by telephone, either directly or through a family member or general practitioner. For patients who died during follow-up, hospital records and necropsy data were reviewed.

The three primary endpoints were all-cause mortality, MACE, and hospitalization for heart failure. MACE was defined as the composite of cardiovascular death, non-fatal myocardial infarction, new revascularization (PCI or CABG), and stroke or transient ischemic attack; the time of the earliest component event was used in time-to-event analyses. Hospitalization for heart failure was defined as the first post-discharge admission with a primary diagnosis of acute decompensated heart failure, supported by clinical signs and symptoms, elevated natriuretic peptides, and/or radiographic evidence of pulmonary congestion. Heart failure events that occurred during the index STEMI hospitalization were not counted as follow-up events; only the first post-discharge episode was included in the time-to-event analysis. Secondary endpoints were the individual components of MACE, analyzed separately, and target vessel revascularization. New-onset diabetes during follow-up—recorded as an exploratory outcome—was defined as a new clinical diagnosis of type 2 diabetes documented at any follow-up visit in a patient who had no diabetes at the index event.

Follow-up time was calculated in months from the index pPCI procedure. For patients without an event, follow-up was administratively censored at 120 months.

### 2.5. Statistical Analysis

Continuous variables were expressed as mean ± SD, and categorical variables as counts (percentages). Between-group comparisons used Student’s *t*-test for continuous variables and the χ^2^ test (or Fisher’s exact test when expected cell counts were below 5) for categorical variables. Within-group changes in medication use between the 1-month and 60-month follow-ups were assessed with McNemar’s test. Statistical significance was defined as two-sided *p* < 0.05. The number of missing values is reported separately for each variable; missing values were not imputed for descriptive comparisons, and the multivariable Cox models used complete case analysis. Descriptive analyses and Cox regression were performed in IBM SPSS Statistics version 20 (IBM, Armonk, NY, USA).

Cumulative event rates were estimated using the Kaplan–Meier method (1 − S(t)) and compared with the log-rank test. Number-at-risk tables are shown beneath each survival curve. Independent predictors of each primary endpoint were identified using multivariable Cox proportional-hazards regression, adjusted for sex, age, LVEF, previous myocardial infarction, Killip class ≥ 2, MetS status, and multivessel coronary disease—variables selected a priori on the basis of clinical relevance and the prior literature. The proportional hazards assumption was assessed graphically using log-minus-log plots and statistically using Schoenfeld residuals. Hazard ratios are reported with 95% confidence intervals. The SYNTAX score was not included in the primary models to avoid collinearity with multivessel disease but was examined separately by Spearman correlation and SYNTAX tertile survival analysis.

Two additional sensitivity analyses were performed for the heart failure endpoint to account for the competing risk of death. The cumulative incidence function was estimated using the Aalen–Johansen method, and a Fine–Gray subdistribution hazard Cox model was fitted with inverse probability of censoring weights, following Geskus [17]. Bootstrap 95% confidence intervals (1000 resamples) are reported for the cumulative incidence at ten years.

The dose–response relationship between the number of MetS criteria met and each outcome was assessed using Spearman’s rank correlation, both for the full cohort and the subset of MetS-positive patients (MetS criteria range: 3–5). For the obesity paradox analysis, obesity was defined using the same waist circumference thresholds as those used in the MetS definition, and the interaction between MetS and obesity was examined using a stratified Kaplan–Meier analysis. New-onset diabetes was compared across MetS groups using a χ^2^ test.

### 2.6. Machine Learning Analysis

To complement the Cox regression and provide patient-level interpretation, four machine learning classifiers were trained to predict each ten-year binary outcome using a feature set of 16 clinical variables available at or shortly after the index hospitalization: logistic regression (with L2 regularization, C = 0.1), random forest (300 trees, max depth 5), gradient boosting (200 estimators, max depth 3, learning rate 0.05) and an XGBoost equivalent gradient boosting model (500 estimators, max depth 4, learning rate 0.03, row and column subsampling 0.8). Models were evaluated using 10-fold stratified cross-validation. Missing feature values were imputed with the median within each training fold to prevent data leakage. Discrimination was assessed using AUC-ROC, and calibration was assessed using the Brier score and graphical calibration curves; observed event rates were also compared across quartiles of predicted probability. All ML analyses were implemented using scikit-learn 1.8, NumPy, pandas, and SciPy.

Feature importance was quantified in two complementary ways. For the gradient boosting models, the mean decrease in impurity (Gini importance) was averaged across the random forest and gradient boosting models. For the XGBoost equivalent model, SHAP (SHapley Additive exPlanations) values were computed using the TreeSHAP algorithm from the ‘shap’ Python library (version 0.46) on a stratified random subsample of 200 patients (random seed 42). To assess the robustness of feature rankings, 95% confidence intervals for each feature’s mean absolute SHAP value were derived from 1000 bootstrap resamples.

To address the binary outcome simplification in the SHAP analysis, survival-aware sensitivity analysis was performed using a Random Survival Forest [18], with 200 trees, a minimum of 10 samples per leaf, and √*p* random feature sampling at each split. Discrimination was assessed using Harrell’s concordance index in 10-fold cross-validation, and feature importance was quantified by permutation; each feature was permuted in turn, and the resulting drop in the c-index was recorded.

### 2.7. Reproducibility and Data Availability

All random number generators used in the analysis were seeded with random_state = 42. The analytical dataset contains direct patient identifiers and cannot be shared publicly under institutional policy and the Serbian Law on Personal Data Protection (Official Gazette RS, No. 87/2018). De-identified summary statistics and the complete analysis code are available from the corresponding author upon reasonable request.

## 3. Results

### 3.1. Baseline Characteristics

Of the 506 patients in the analytical sample (Figure S1, Supplementary Material), 216 (42.7%) met three or more AHA/NHLBI criteria and were classified as MetS-positive; the remaining 290 (57.3%) were negative. The two groups are compared in Table 1.

MetS-positive patients were, on average, three years older (60.7 ± 11.5 vs. 57.7 ± 10.8 years, *p* = 0.004) and had a higher proportion of women (26.9% vs. 17.8%, *p* = 0.012). By contrast, smoking was more prevalent among MetS-negative patients (76.9% vs. 66.2%, *p* = 0.007). Family history of CVD, prior myocardial infarction, and previous PCI, COPD, or renal insufficiency did not differ significantly between groups. By design, all five individual MetS components were more prevalent in the MetS-positive group (all *p* < 0.001). Multivessel coronary disease was significantly more common in MetS-positive patients (70.4% vs. 61.2%, *p* = 0.033). The angiographic and procedural variables—infarct territory, culprit artery, symptom-to-balloon time, stent characteristics, and rate of multivessel intervention at index procedure—were comparable between groups, as were peak CK, peak CK-MB, and admission LVEF (49.6 ± 9.6% vs. 49.7 ± 10.1%, *p* = 0.974). The SYNTAX score, available in 464 of 506 patients (91.7%), was significantly higher in the MetS-positive group (19.10 ± 6.76 vs. 17.34 ± 6.83, *p* = 0.006). Peak CK-MB was missing in 65.4% of cases (reflecting a transition in laboratory protocols during the enrollment period) and was therefore excluded from the multivariable models.

### 3.2. Medication Use During Follow-Up

Use of evidence-based secondary prevention medications at the first follow-up visit (month 1) and at the 60-month visit is shown in Figure S2 (Supplementary Material). At month 1, aspirin and beta-blocker use was high and similar between groups (aspirin 92% vs. 91%, *p* = 0.682; beta-blocker 82% vs. 79%, *p* = 0.532), as was statin use (88% vs. 84%, *p* = 0.405). RAAS-blocker use was significantly higher in MetS-positive patients (79% vs. 69%, *p* = 0.019), consistent with the higher prevalence of hypertension. By month 60, aspirin and beta-blocker use had declined to a comparable extent in both groups (aspirin 71% vs. 76%, *p* = 0.208; beta-blocker 63% vs. 65%, *p* = 0.675). Statin use declined more sharply in the MetS-negative group (from 84.5% to 74.1%; McNemar *p* < 0.001) than in the MetS-positive group (from 88% to 72%); the between-group difference at month 60 was borderline significant (*p* = 0.063).

### 3.3. Adverse Events During Ten-Year Follow-Up

Cumulative incidence rates and incidence rates per 100 person-years for all adverse events are shown in Table 2. All-cause mortality occurred in 64 MetS-positive patients (29.6%, 3.67 events per 100 person-years) compared with 65 MetS-negative patients (22.7%, 2.70 per 100 person-years; *p* = 0.080). Cardiovascular mortality followed the same pattern (25.0% vs. 18.5%, *p* = 0.080). Non-fatal myocardial reinfarction was significantly more frequent in the MetS-positive group (12.5% vs. 5.6%, *p* = 0.006), as were new revascularization (20.4% vs. 10.8%, *p* = 0.003) and target vessel revascularization (12.5% vs. 4.9%, *p* = 0.002). The most pronounced between-group difference was observed for hospitalization due to heart failure with 36 MetS-positive patients (16.7%, 2.22 events per 100 person-years) versus 17 MetS-negative patients (5.9%, 0.71 per 100 person-years; *p* < 0.001)—a more than threefold difference in incidence rate.

### 3.4. Kaplan–Meier Survival Analysis

#### 3.4.1. Primary Outcomes by MetS Status

**Figure 1:**
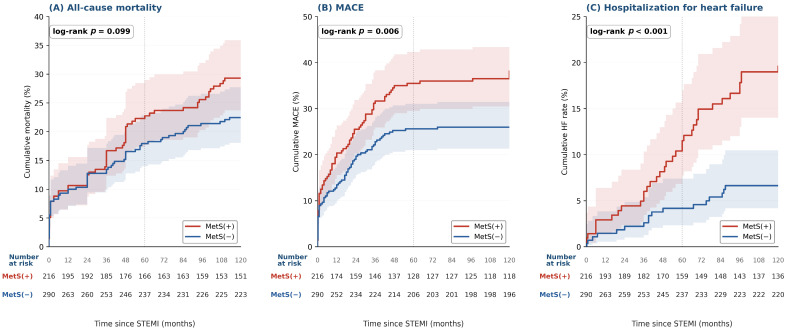
Kaplan–Meier cumulative event rate curves for the three primary outcomes. Cumulative event rates [1 − S(t)] for all-cause mortality (**A**), MACE (**B**), and hospitalization for heart failure (**C**), stratified by metabolic syndrome status. Shaded bands show 95% pointwise confidence intervals; the vertical dotted line marks the 60-month timepoint. *p*-values from the log-rank test are shown in each panel. Number-at-risk tables are shown beneath each panel for both groups. Abbreviations: MetS—metabolic syndrome; MACE—major adverse cardiovascular event; HF—heart failure.

Cumulative event rate curves for the three primary endpoints are shown in Figure 1, with number-at-risk tables beneath each panel. At ten years, cumulative mortality was 29% in MetS-positive patients versus 22.5% in MetS-negative patients (Figure 1A); the log-rank test was not significant (*p* = 0.099). MetS-positive patients accumulated MACE more rapidly than MetS-negative patients, reaching 37% versus 26% at ten years (Figure 1B; log-rank *p* = 0.006). The largest separation was observed for heart failure, with rates of 19.6% in MetS-positive versus 6.6% in MetS-negative patients (Figure 1C; log-rank *p* < 0.001). The number-at-risk tables confirm that follow-up density was maintained in both groups throughout the 120-month observation window.

#### 3.4.2. Outcomes by Number of MetS Criteria Fulfilled

**Figure 2:**
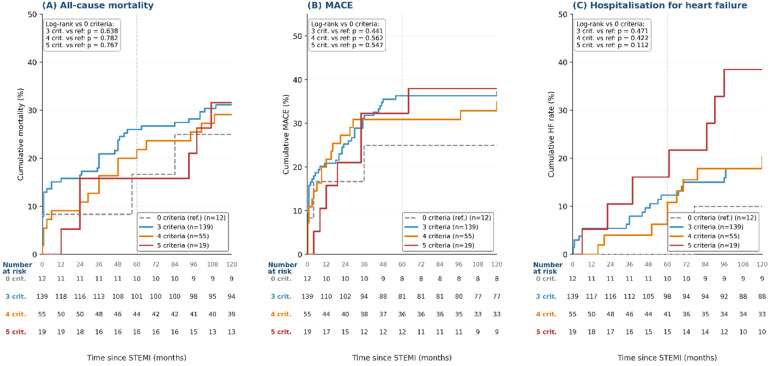
Kaplan–Meier curves stratified by the number of metabolic syndrome criteria met. Cumulative event rate curves for all-cause mortality (**A**), MACE (**B**), and heart failure hospitalization (**C**), stratified by the number of MetS criteria met (3, 4, or 5) and compared with the reference group of patients with no criteria met (dashed gray line, n = 12). Log-rank *p*-values for each subgroup versus the reference group are shown in each panel. Number-at-risk tables for all four subgroups are shown beneath each panel. Abbreviations as in Figure 1.

Patients were stratified by the number of MetS criteria met (3, 4, or 5) and compared with a reference group of patients with no criteria met (n = 12). Results are shown in Figure 2. None of the three subgroups differed significantly from the no-MetS reference group for mortality (3 vs. ref *p* = 0.638; 4 vs. ref *p* = 0.782; 5 vs. ref *p* = 0.767) or for MACE (3 vs. ref *p* = 0.443; 4 vs. ref *p* = 0.558; 5 vs. ref *p* = 0.566). For heart failure, the five-criteria subgroup showed a steep increase in incidence after month 72, reaching 38% at ten years; this did not reach statistical significance compared with the small reference group (*p* = 0.086, with the three- and four-criteria subgroups both *p* > 0.4).

### 3.5. Multivariable Cox Proportional-Hazards Regression

Cox models, adjusted for sex, age, LVEF, previous myocardial infarction, Killip class ≥ 2, MetS status, and multivessel coronary disease, are summarized in Figure S3 (Supplementary Material).

#### 3.5.1. All-Cause Mortality

MetS was not an independent predictor of all-cause mortality (HR 1.092, 95% CI 0.741–1.610, *p* = 0.657). Independent predictors included age (HR 1.068 per year, 95% CI 1.048–1.088, *p* < 0.001), LVEF (HR 0.963 per percentage point, 95% CI 0.940–0.986, *p* = 0.002), previous myocardial infarction (HR 1.845, 95% CI 1.171–2.907, *p* = 0.008), and Killip class ≥ 2 at admission (HR 2.565, 95% CI 1.613–4.080, *p* < 0.001). Sex and multivessel disease were not significant predictors.

#### 3.5.2. MACE

MetS was an independent predictor of MACE (HR 1.473, 95% CI 1.043–2.081, *p* = 0.028). Other independent predictors were age (HR 1.034 per year, *p* < 0.001), LVEF (HR 0.961 per percentage point, *p* < 0.001), and Killip class ≥ 2 (HR 1.782, *p* = 0.009). Previous MI, sex, and multivessel disease were not significant predictors.

#### 3.5.3. Heart Failure

MetS was the strongest independent predictor of heart failure (HR 2.860, 95% CI 1.568–5.215, *p* < 0.001). The only other predictor that reached significance was reduced LVEF (HR 0.914 per percentage point, 95% CI 0.881–0.948, *p* < 0.001). Killip class, previous MI, age, sex, and multivessel disease did not reach significance.

#### 3.5.4. Sensitivity Analysis: Heart Failure Under Competing Risk of Death

**Figure 3:**
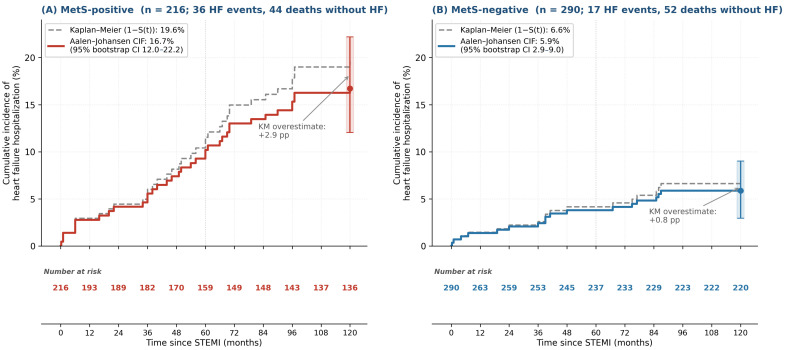
Aalen–Johansen cumulative incidence function (CIF) for heart failure hospitalization under the competing risk of death: (**A**) MetS-positive (n = 216); (**B**) MetS-negative (n = 290). Solid lines, Aalen–Johansen CIF; dashed gray lines, naive Kaplan–Meier estimator (1 − S(t)) for comparison. Error bars at 10 years represent 95% bootstrap confidence intervals (1000 resamples). Number-at-risk tables are shown beneath each panel.

Because 96 of 506 patients (19.0%) died without a preceding heart failure event, the standard Cox model—which treats deaths as standard censoring—may overestimate cumulative heart failure incidence. We therefore performed a sensitivity analysis using the Aalen–Johansen estimator for the cumulative incidence function (CIF) and a Fine–Gray subdistribution hazard Cox model with inverse probability of censoring weights. The Aalen–Johansen ten-year CIF was 16.7% (95% bootstrap CI, 12.0–22.2%) in MetS-positive and 5.9% (95% bootstrap CI, 2.9–9.0%) in MetS-negative patients (Figure 3); the naive Kaplan–Meier estimator overestimated the absolute incidence by 2.9 and 0.8 percentage points, respectively. The Fine–Gray subdistribution hazard ratio for MetS was 2.61 (95% CI 1.44–4.75, *p* = 0.002)—virtually identical to the cause-specific hazard ratio of 2.86 (slightly attenuated, as expected). The conclusion that MetS is a strong independent predictor of heart failure is therefore robust in competing risk treatment.

### 3.6. Additional Analyses (Supplementary Material)

#### 3.6.1. SYNTAX Score and Coronary Atherosclerotic Burden (Supplementary Figure S4)

When patients were stratified into SYNTAX score tertiles (low ≤ 14, n = 155; mid 15–22, n = 166; high ≥ 23, n = 142), a clear gradient in ten-year event rates emerged across all three outcomes: MACE rose from 14% to 27% to 51%; mortality from 10% to 23% to 42%; and heart failure from 4% to 13% to 15%. The SYNTAX score showed a positive correlation with the ten-year MACE rate (Spearman ρ = 0.337, *p* < 0.001).

#### 3.6.2. Obesity Paradox (Supplementary Figure S5)

Among non-obese patients (MetS-positive n = 35; MetS-negative n = 109), MetS-positive status was associated with significantly higher 10-year mortality (42.9% vs. 21.1%; log-rank *p* = 0.008). Among obese patients (n = 181 in each group), the difference in mortality was no longer significant (26.5% vs. 23.2%; log-rank *p* = 0.529). The excess mortality attributable to MetS was 21.8 percentage points among non-obese patients and only 3.3 points among obese patients. For MACE and heart failure, MetS retained a meaningful excess risk in both subgroups (MACE excess: +16.4% non-obese vs. +12.2% obese; HF excess: +13.7% vs. +11.6%).

#### 3.6.3. MetS Criteria Burden and New-Onset Diabetes (Supplementary Figure S6)

When stratified by the number of fulfilled MetS criteria (0–5), heart failure incidence rose from 8.3% at zero criteria to 36.8% at five criteria. Among MetS-positive patients (3–5 criteria), the dose–response was modest for heart failure (Spearman ρ = 0.106, *p* = 0.123) and absent for MACE and mortality (ρ ≈ −0.01 for both, *p* > 0.8). New-onset diabetes during follow-up occurred in 17 MetS-positive patients (7.9%) versus eight MetS-negative patients (2.8%, χ^2^ *p* = 0.016).

### 3.7. Machine Learning Performance and SHAP Analysis

#### 3.7.1. Model Performance

The four machine learning models—logistic regression (with L2 regularization), random forest, gradient boosting, and an XGBoost equivalent gradient boosting classifier—were evaluated using 10-fold stratified cross-validation. Receiver operating characteristic (ROC) curves are shown in Supplementary Figure S7; calibration curves for the best-performing model at each endpoint are shown in Supplementary Figure S8; and a complete summary of cross-validated discrimination and calibration metrics is provided in Supplementary Table S1.

For all-cause mortality, all four models showed similar discrimination, with cross-validated AUCs clustering between 0.825 and 0.834 (logistic regression 0.825, random forest 0.827, XGBoost equivalent 0.829, gradient boosting 0.834). For MACE, the four models performed comparably, with random forest achieving the highest AUC (0.752), followed by logistic regression (0.747), XGBoost equivalent (0.721), and gradient boosting (0.710). For heart failure, logistic regression achieved the highest discrimination (AUC 0.793), followed by random forest (0.775), XGBoost equivalent (0.733), and gradient boosting (0.715).

Calibration was assessed using quantile binned plots of observed versus mean-predicted probabilities for the best-performing model for each endpoint (Supplementary Figure S8). Brier scores confirmed adequate probabilistic calibration: mortality 0.126 (gradient boosting), MACE 0.175 (random forest), and heart failure 0.082 (random forest; Brier; logistic regression: AUC, Brier 0.083). The mortality model, in particular, showed near-ideal calibration across the full range of predicted probabilities.

Risk quartile stratification (Supplementary Figure S9) showed a marked monotonic gradient in observed event rates from the lowest- to the highest-risk quartile, supporting the clinical utility of the predicted scores. For mortality, observed ten-year event rates rose from 5.5% in Q1 to 64.6% in Q4 (an 11.7-fold increase). For MACE, rates rose from 11.0% to 56.7% (a 5.1-fold increase). For heart failure, rates rose from 1.6% to 26.0% (16.5-fold), the steepest relative gradient across the three endpoints.

#### 3.7.2. Feature Importance

Tree-based feature importance (mean decrease in impurity, normalized within each model and averaged across the random forest and gradient boosting classifiers, with 95% bootstrap confidence intervals from 100 resamples) is shown in Supplementary Figure S9. The leading features differ markedly across the three endpoints, providing convergent evidence with the SHAP and Random Survival Forest analyses.

For MACE, the SYNTAX score was the top-ranked feature (averaged Gini 0.190, 95% CI 0.162–0.220), followed by age (0.133, 95% CI 0.107–0.162), Killip class ≥ 2 (0.130, 95% CI 0.087–0.149), peak CK (0.118, 95% CI 0.102–0.133), and LVEF (0.102, 95% CI 0.087–0.125). For mortality, age dominated by a wide margin (0.278, 95% CI 0.245–0.309), followed by Killip class ≥ 2 (0.168, 95% CI 0.120–0.195) and peak CK (0.092, 95% CI 0.080–0.105). For both endpoints, MetS-related features ranked in the lower half of the importance hierarchy; for MACE, the cumulative number of MetS criteria reached an averaged Gini of 0.024, and binary MetS 0.012, while for mortality, both metrics fell below 0.025.

For heart failure, the picture changed. LVEF was the top-ranked feature (averaged Gini 0.200, 95% CI 0.159–0.219), followed by peak CK (0.120, 95% CI 0.097–0.150) and the cumulative number of MetS criteria (0.118, 95% CI 0.092–0.148)—the latter outranking established prognostic markers including WMSI (0.096), age (0.084), and SYNTAX score (0.080). The binary MetS classification, by contrast, contributed only a modest Gini value of approximately 0.029, which is approximately fourfold below the continuous criteria count metric.

#### 3.7.3. SHAP Explainability Analysis

**Figure 4:**
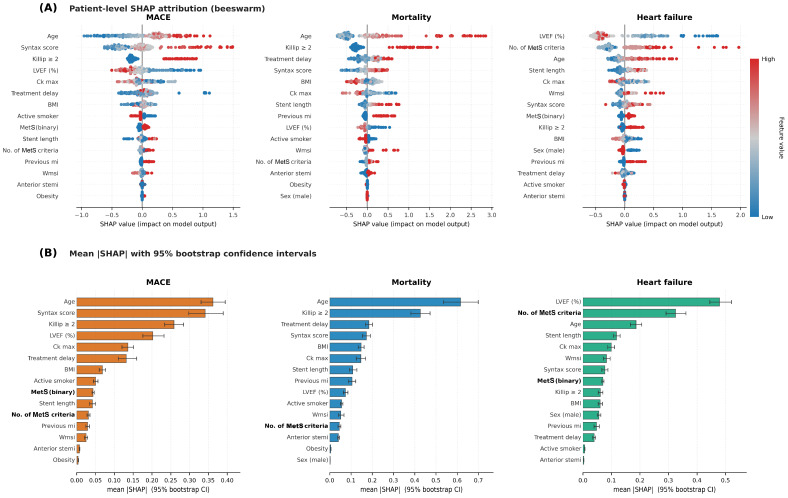
SHAP attribution analysis of the XGBoost equivalent classifier for the three primary ten-year endpoints. Top 15 features are shown for each endpoint, ranked by mean absolute SHAP value computed on a stratified random subsample of 200 patients (random seed 42). (**A**) Beeswarm plots display the distribution of patient-level SHAP values; each dot represents one patient, with horizontal position indicating the magnitude and direction of the feature’s contribution to the model prediction. Color encodes the original feature value (red, high; blue, low; and gray, intermediate or missing), revealing the direction of the relationship between each feature and the predicted probability. (**B**) Mean absolute SHAP values with 95% bootstrap confidence intervals (1000 resamples), quantifying the overall importance of each feature and providing a measure of statistical uncertainty. MetS-related features are shown in bold.

SHAP attribution was computed using the TreeSHAP algorithm from the standard ‘shap’ Python library (version 0.46) on a stratified random subsample of 200 patients. Beeswarm plots showing the distribution of patient-level SHAP values for the top 15 features and the corresponding mean absolute SHAP values with 95% bootstrap confidence intervals (1000 resamples) are shown in Figure 4 (panels A and B, respectively). To address potential ambiguity in feature ordering between panels, each panel is sorted independently by the mean |SHAP| value within its endpoint and retains its own feature labels.

For MACE prediction, age and SYNTAX score were the two features with the highest patient-level contribution (mean |SHAP| 0.36 and 0.34, with bootstrap 95% CIs cleanly separated from the rest of the feature set), followed by Killip class ≥ 2 (0.26) and LVEF (0.20). MetS-related features ranked in the lower half of the importance hierarchy (number of MetS criteria 0.03; binary MetS 0.04). For mortality, age dominated by a wide margin (mean |SHAP| 0.62, 95% CI 0.53–0.70), followed by Killip class (0.43), treatment delay (0.18), and SYNTAX score (0.17); MetS-related features ranked near the bottom (binary MetS effectively at zero; number of MetS criteria 0.05).

For heart failure, the two features with the highest mean |SHAP| values were LVEF (mean |SHAP| 0.48, 95% CI 0.44–0.52) and the cumulative number of MetS criteria (0.32, 95% CI 0.29–0.36)—the only two features whose bootstrap CIs cleanly separated from the rest of the set. The number of MetS criteria outperformed the binary MetS diagnosis (0.07) by about fivefold, indicating that the cumulative number of MetS criteria contributes more to the model output than the threshold-based binary classification. The corresponding beeswarm panel (Figure 4A, right) shows that patients with a high number of MetS criteria are systematically shifted to the right of zero, reflecting consistently higher predicted heart failure probability, while patients with few or no MetS criteria cluster near zero or to the left.

#### 3.7.4. Sensitivity Analysis: Survival-Aware Machine Learning

**Figure 5:**
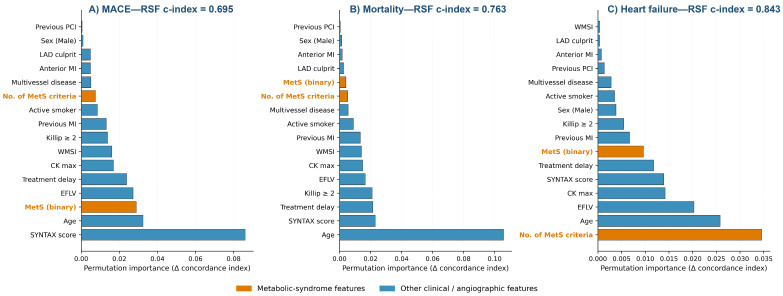
Random Survival Forest permutation feature importance. Permutation importance for MACE (**A**), all-cause mortality (**B**), and heart failure hospitalization (**C**), reported as the drop in the concordance index when each feature is individually permuted in a Random Survival Forest fitted to the full analytical sample. Metabolic syndrome features are shown in orange. The number of MetS criteria ranks first for heart failure prediction (Δc-index = +0.035), providing convergent evidence with the SHAP analysis (Figure 4B, where MetS criteria ranked second by mean |SHAP|). The cross-validated Harrell’s c-index for each outcome is shown in the panel header. Abbreviations as in Figure 4.

Because the SHAP analysis treats ten-year outcomes as binary classification targets and does not account for censoring, we performed a sensitivity analysis using a Random Survival Forest with permutation feature importance. The model achieved cross-validated Harrell’s c-index of 0.763 ± 0.106 for mortality, 0.695 ± 0.083 for MACE, and 0.843 ± 0.084 for heart failure (Figure 5)—the highest discrimination among the machine learning approaches for the heart failure outcome. Permutation importance ranked the cumulative number of MetS criteria first for heart failure (Δc-index = +0.035), surpassing age (+0.026), LVEF (+0.020), peak CK (+0.014), and SYNTAX score (+0.014). For mortality, age dominated permutation importance (Δc-index = +0.106); for MACE, SYNTAX score (+0.086) and age (+0.032) ranked first and second.

The three feature attribution frameworks produced concordant rankings for heart failure: the cumulative number of MetS criteria ranked third by averaged Gini importance (Section 3.7.2), second by mean |SHAP| (Section 3.7.3), and first by Random Survival Forest permutation importance (Section 3.7.4). The binary MetS diagnosis ranked outside the top five features in all three frameworks.

## 4. Discussion

We followed 506 STEMI patients treated with primary PCI for ten years, stratified by AHA/NHLBI metabolic syndrome status. The principal findings are summarized as follows. Metabolic syndrome was not an independent predictor of all-cause or cardiovascular mortality at ten years, despite numerically higher rates in the MetS-positive group. However, it was an independent predictor of MACE (HR 1.473, *p* = 0.028), driven primarily by recurrent myocardial infarction and target-vessel revascularization. Most importantly, MetS was a strong independent predictor of hospitalization for heart failure (cause-specific HR 2.86, Fine–Gray subdistribution HR 2.61, both *p* < 0.005), with reduced LVEF as the only co-significant predictor. The excess mortality risk associated with MetS was selectively attenuated by obesity, providing empirical support for an obesity paradox specific to the MetS phenotype in STEMI. Two methodologically independent machine learning attribution frameworks—TreeSHAP for a binary classification XGBoost model and permutation importance for a survival aware Random Survival Forest—converged on the cumulative number of MetS criteria, rather than the binary diagnosis, as the leading individual-level contributor to heart failure risk in this cohort.

The independent association between MetS and ten-year MACE (HR 1.473) extends our four-year analysis from the same cohort, which yielded a stronger HR of 1.834 [13]. The attenuation over time is biologically coherent, as MetS predominantly accelerates early atherosclerotic progression and inflammatory restenosis, mechanisms that are most active in the first five years after revascularization, while age and left ventricular function increasingly dominate the prognosis later. The mechanism is supported by direct anatomical evidence—MetS-positive patients had a significantly higher SYNTAX score (19.10 ± 6.76 vs. 17.34 ± 6.83, *p* = 0.006), with a strong positive Spearman correlation between SYNTAX and ten-year MACE (ρ = 0.337, *p* < 0.001). This is consistent with the well-established role of insulin-resistance-driven inflammation, NLRP3 inflammasome activation, and a prothrombotic state in promoting plaque vulnerability and in-stent restenosis [5]. The convergence of two ML frameworks reinforces this conclusion: TreeSHAP ranked age and SYNTAX score as the dominant patient-level features for MACE (mean |SHAP| 0.36 and 0.34), and the Random Survival Forest reached the same conclusion via permutation importance. For ischemic recurrence, anatomical complexity of established coronary disease—which MetS helps create—is the proximate determinant; MetS itself contributes to MACE primarily by promoting more severe baseline anatomy.

The failure of MetS to independently predict mortality at ten years—despite numerically higher rates in the MetS-positive group (29.6% vs. 22.7%)—is among the most contentious findings in the literature, and our result aligns with the majority of published cohorts. Won et al. found no significant effect on long-term survival in 1100 STEMI patients with drug-eluting stents [7], while Geraiely et al., in a propensity score-matched analysis of 2651 pPCI patients, found no association between MetS and one-year mortality [8]. Dohi et al. observed mortality curves diverging only after five years [9], a pattern which was also visible in our data, though not reaching statistical significance, raising the possibility that even longer follow-up could ultimately reveal a mortality signal.

Several non-mutually exclusive hypotheses may help explain this null mortality finding. Medication confounding is plausible: the MetS-negative group experienced a sharper decline in statin use from 84.5% at month 1 to 74.1% at month 60 (McNemar *p* < 0.001) than the MetS-positive group. The FAST-STEMI registry showed that statin discontinuation within six months after STEMI was independently associated with more than a twofold increase in cardiovascular and all-cause mortality [19], so suboptimal long-term adherence in the MetS-negative group may have selectively eroded their survival advantage. The independent predictors of mortality in our Cox model—age, LVEF, previous MI, and Killip class—are acute hemodynamic and structural parameters that dominate prognosis irrespective of metabolic status; this is consistent with the CAMI registry XGBoost/SHAP analysis [20] and with our own SHAP results, in which age (mean |SHAP| 0.62) contributed more than any other feature to mortality. We emphasize that these are interpretive hypotheses consistent with our observations rather than causal conclusions, which an observational design cannot establish.

The obesity paradox offers a second, complementary explanation. Among non-obese patients, MetS-positive status was associated with significantly higher ten-year mortality (42.9% vs. 21.1%, *p* = 0.008), whereas among obese patients the difference disappeared (26.5% vs. 23.2%, *p* = 0.529). The 21.8 percentage point excess mortality among non-obese MetS-positive patients fell to 3.3 points in the obese stratum. The 2023 meta-analysis by Wang et al. confirmed lower in-hospital and long-term mortality among obese STEMI patients [21], and Şaylık et al. reached the same conclusion in a comprehensive meta-analysis of ACS [22]. Our data add a previously unreported dimension: the obesity paradox in STEMI is not uniform but is specifically operative among patients with MetS. The lean MetS phenotype—characterized by visceral adiposity, severe insulin resistance, and unfavorable adipokine profiles despite an unremarkable BMI—may carry a disproportionate share of the prognostic burden of metabolic dysregulation in this stratum. The likely biological mediator is adiponectin, whose levels are relatively preserved in obese MetS patients, which exerts direct cardioprotective and anti-inflammatory effects [23]. Crucially, the paradox was selective, as MetS retained meaningful excess risk for both MACE (excess +16.4% non-obese vs. +12.2% obese) and heart failure (+13.7% vs. +11.6%) in both obesity strata, so the survival benefit of obesity attenuates the inflammatory pathways driving early mortality without reducing the anatomical or metabolic cardiomyopathy pathways.

The strongest finding of this study is that MetS nearly triples the cause-specific hazard of heart failure hospitalization (HR 2.86, 95% CI 1.57–5.22, *p* < 0.001). This conclusion is robust in competing risk treatment: the Fine–Gray subdistribution hazard model yielded an essentially identical HR of 2.61 (95% CI 1.44–4.75, *p* = 0.002). The Aalen–Johansen cumulative incidence at ten years was 16.7% in MetS-positive and 5.9% in MetS-negative patients, with the naive Kaplan–Meier estimator overestimating absolute incidence by 2.9 and 0.8 percentage points, respectively. The strength and persistence of this association—in patients with virtually identical admission LVEF (49.6% vs. 49.7%, *p* = 0.974)—implicates pathophysiological mechanisms beyond the acute infarction and points to the metabolic cardiomyopathy that characterizes the MetS phenotype.

The pathophysiology is well characterized. The primary mediator is insulin resistance, which impairs myocardial glucose uptake and forces cardiomyocytes to shift to fatty acid oxidation—a less metabolically efficient substrate that increases oxygen consumption, generates reactive oxygen species, and progressively impairs mitochondrial function [24]. Using ^18^F-FDG PET-MR in 821 asymptomatic individuals from the PESA cohort, Succurro et al. directly demonstrated that MetS traits are associated with a significantly lower insulin-stimulated myocardial glucose metabolic rate—the earliest detectable stage of metabolic cardiomyopathy [25]. At the molecular level, MetS induces NLRP3 inflammasome activation, NF-κB signaling, and AGE–RAGE-mediated myocardial fibrosis [5,25], collectively promoting interstitial fibrosis, cardiomyocyte hypertrophy, microvascular dysfunction, and the clinical phenotype of HFpEF. The significantly higher rate of new-onset diabetes during follow-up in MetS-positive patients (7.9% vs. 2.8%, *p* = 0.016) likely contributes to the late-divergence pattern of heart failure incidence visible after month 72 in our Kaplan–Meier curves.

Three methodologically independent attribution frameworks identified the cumulative number of MetS criteria—rather than the binary diagnosis—as one of the top three individual-level drivers of heart failure risk. In the TreeSHAP analysis of the XGBoost binary classifier, the number of MetS criteria ranked second only to LVEF (mean |SHAP| 0.32 vs. 0.48; bootstrap 95% CIs cleanly separated from the rest of the feature set) and was approximately fivefold higher than the contribution of the binary MetS diagnosis (0.07). To address the binary outcome simplification of the SHAP analysis, a survival-aware Random Survival Forest with permutation importance was used: in this analysis, the number of MetS criteria emerged as the top-ranked feature for heart failure (Δc-index = +0.035), surpassing LVEF, age, and SYNTAX score, with the RSF achieving the highest cross-validated discrimination of any ML model (Harrell’s c-index 0.843). The averaged Gini importance across the random forest and gradient boosting classifiers ranked the cumulative criteria count third for heart failure (Gini 0.118, 95% CI 0.092–0.148), behind LVEF and peak CK only, and approximately fourfold higher than the binary MetS classification.

The convergence across three distinct frameworks—together with the consistency with the Cox HR of 2.86—is compatible with a continuous burden interpretation of metabolic risk rather than a binary-threshold interpretation of heart failure risk after STEMI; the binary MetS diagnosis ranked outside the top five features in all three frameworks. To our knowledge, this is the first patient-level, model-agnostic demonstration of the continuous nature of the MetS–HF dose–response relationship in a long-term STEMI cohort. We emphasize, however, that MetS status was assessed only at admission, so our findings reflect the prognostic value of the baseline metabolic profile rather than evidence that prospective modification of individual MetS components reduces heart failure incidence—a question that requires longitudinal MetS exposure data and dedicated interventional trials.

The clinical implications of this study are actionable but constrained by the limits of an observational design. The identification of MetS as an independent predictor of both MACE and heart failure over ten years supports systematic metabolic assessment of all STEMI survivors using AHA/NHLBI criteria before discharge. The obesity paradox has a specific implication for risk stratification, where algorithms based on the binary MetS classification will overestimate mortality risk in obese MetS-positive patients and, more critically, underestimate it in lean MetS-positive patients, who accounted for 42.9% of ten-year mortality in our cohort. The pattern we observed in the non-obese stratum is consistent with the lean cardiometabolic phenotype described in the obesity paradox literature; lean MetS-positive patients may warrant closer follow-up in dedicated studies, although our subgroup numbers were small and this observation should be regarded as exploratory. Convergent ML evidence that the number of metabolic criteria, rather than the binary diagnosis, drives the heart failure signal is consistent with the hypothesis that patients with more concurrent metabolic abnormalities at baseline carry incrementally higher long-term heart failure risk; whether prospective modification of individual MetS components reduces this risk cannot be answered by our data and warrants dedicated interventional studies. The present cohort predates the routine secondary-prevention use of SGLT2 inhibitors and GLP-1 receptor agonists; whether these therapies, which have since acquired class 1A indications for HFpEF in the 2023 ESC Heart Failure guidelines [26], modify ten-year heart failure incidence in STEMI survivors with high baseline metabolic burden remains a hypothesis for dedicated randomized trials.

This study has several limitations. It is a single-center, prospective, observational cohort study at a Serbian tertiary academic institution, limiting generalizability to other populations and healthcare systems; bare-metal stents predominated, reflecting the 2009–2010 standard of care, and contemporary drug-eluting stents would likely attenuate the revascularization component of MACE. MetS was defined only at admission—the acute phase of STEMI can transiently elevate glucose, triglycerides, and blood pressure, potentially overclassifying some patients—and longitudinal MetS exposure could not be quantified; consequently, our findings on the cumulative number of MetS criteria as a predictor of heart failure should be interpreted as reflecting the prognostic value of the baseline metabolic profile, not as evidence that modifying individual components during follow-up reduces incidence. Medication adherence data were available only at months 1 and 60, so continuous adherence and the time-dependent introduction of novel agents (P2Y12 inhibitors, SGLT2 inhibitors, GLP-1 receptor agonists) represent residual confounding inherent to a long-term observational design. Several potentially relevant predictors were not captured (serial LVEF, NT-proBNP, diastolic function indices, adipokines), and the subgroup meeting all five MetS criteria (n = 19) was too small to support firm conclusions about the extreme end of metabolic burden. With respect to the survival analyses, the Fine–Gray sensitivity analysis confirmed that competing-risk effects modestly inflate naive heart failure incidence estimates without altering the principal conclusion. The machine learning analysis was performed on a single cohort with stratified 10-fold internal cross-validation but without external geographic validation; the models should therefore be interpreted as tools for feature attribution and hypothesis generation rather than deployable clinical risk calculators. Finally, no formal multiplicity correction was applied across the family of secondary endpoints, which may inflate the type I error for those comparisons; the three primary endpoints (mortality, MACE, heart failure) were pre-specified and should be interpreted with the highest weight.

## 5. Conclusions

In a single-center prospective cohort of 506 STEMI patients followed for 10 years after primary PCI, metabolic syndrome was a strong independent predictor of hospitalization for heart failure (cause-specific hazard ratio 2.86; Fine–Gray subdistribution hazard ratio 2.61) and a moderate predictor of MACE (HR 1.47), but it did not independently predict all-cause mortality. This null effect was selectively attenuated by obesity, revealing a high-risk lean MetS-positive subgroup that accounted for a disproportionate share of long-term mortality. Across three methodologically independent machine learning attribution frameworks, the cumulative number of MetS criteria—rather than the binary diagnosis—emerged as a leading individual-level contributor to heart failure risk across three independent feature attribution analyses, compatible with a continuous burden interpretation of metabolic syndrome in this clinical context. These observational findings justify systematic baseline metabolic assessment of all STEMI survivors and highlight lean MetS-positive patients as a candidate high-priority subgroup warranting further investigation; whether prospective modification of individual MetS components reduces 10-year heart failure incidence remains a hypothesis to be tested in dedicated interventional studies.

## Figures and Tables

**Table 1:** Baseline characteristics of the analyzed patients.

Parameter	MetS-Positive (n = 216)	MetS-Negative (n = 290)	*p*	Missing, n (%)
**Demographics**				
Age, years (mean ± SD)	60.7 ± 11.5	57.7 ± 10.8	0.004 *	0 (0.0%)
Female sex, n (%)	58 (26.9%)	51 (17.8%)	0.012 *	0 (0.0%)
Cardiovascular risk factors and comorbidities				
History of smoking, n (%)	143 (66.2%)	220 (76.9%)	0.007 *	0 (0.0%)
Family history of CVD, n (%)	103 (47.7%)	126 (44.1%)	0.419	0 (0.0%)
Previous myocardial infarction, n (%)	24 (11.1%)	21 (7.3%)	0.142	0 (0.0%)
Previous PCI, n (%)	15 (6.9%)	11 (3.9%)	0.153	1 (0.2%)
COPD, n (%)	21 (9.7%)	24 (8.4%)	0.605	0 (0.0%)
Renal insufficiency, n (%)	16 (7.4%)	14 (4.9%)	0.240	0 (0.0%)
Metabolic syndrome components				
Abdominal obesity, n (%)	136 (63.0%)	54 (18.9%)	<0.001 *	0 (0.0%)
Hyperglycemia, n (%)	157 (72.7%)	76 (26.6%)	<0.001 *	0 (0.0%)
Hypertriglyceridemia, n (%)	126 (58.3%)	103 (36.0%)	<0.001 *	0 (0.0%)
Hypertension, n (%)	172 (79.6%)	155 (54.2%)	<0.001 *	0 (0.0%)
Low HDL cholesterol, n (%)	113 (52.3%)	114 (39.9%)	<0.001 *	0 (0.0%)
Angiographic and procedural findings				
Anterior infarction, n (%)	87 (40.3%)	135 (47.2%)	0.122	0 (0.0%)
Culprit artery LAD, n (%)	88 (40.7%)	134 (46.9%)	0.172	0 (0.0%)
Multivessel disease, n (%)	152 (70.4%)	175 (61.2%)	0.033 *	0 (0.0%)
Killip class ≥ 2, n (%)	57 (26.4%)	61 (21.3%)	0.181	0 (0.0%)
Symptom-to-balloon time (min)	304 ± 164	296 ± 164	0.600	0 (0.0%)
Number of stents (mean ± SD)	1.51 ± 0.79	1.42 ± 0.80	0.250	0 (0.0%)
Total stent length (mm)	31.6 ± 16.2	30.9 ± 17.1	0.285	10 (2.0%)
Stent diameter (mm)	3.48± 1.92	3.30 ± 1.89	0.708	113 (22.3%)
Bare-metal stents, n (%)	206 (95.4%)	279 (97.5%)	0.181	0 (0.0%)
Multivessel intervention, n (%)	10 (4.6%)	12 (4.2%)	0.814	0 (0.0%)
Laboratory and functional parameters				
LVEF at admission (%)	49.6 ± 9.6	49.7 ± 10.1	0.974	26 (5.1%)
Peak CK (U/L)	2582 ± 1792	2848 ± 1930	0.682	36 (7.1%)
Peak CK-MB (U/L)	227.7 ± 16.0	228.5 ± 18.0	0.942	331 (65.4%)
SYNTAX score	19.10 ± 6.76	17.34 ± 6.83	0.006 *	42 (8.3%)

* *p* < 0.05 (statistically significant). Continuous variables are reported as mean ± standard deviation; categorical variables as n (%). *p*-values are derived from Student’s *t*-test (continuous) or χ^2^ test (categorical). Missing values are reported separately for each variable; they were not imputed for descriptive analyses, and the multivariable Cox models used complete case analysis. Abbreviations: CVD—cardiovascular disease; PCI—percutaneous coronary intervention; COPD—chronic obstructive pulmonary disease; HDL—high-density lipoprotein; LAD—left anterior descending artery; LVEF—left ventricular ejection fraction; CK—creatine kinase; CK-MB—creatine kinase MB isoform; SYNTAX—synergy between percutaneous coronary intervention with taxus and cardiac surgery.

**Table 2:** Adverse events during ten-year follow-up.

Endpoint	MetS-Positive (N = 216) N (%) [Rate Per 100 PY]	MetS-Negative (n = 290) n (%) [Rate Per 100 PY]	*p*-Value	Total Events
All-cause mortality	64 (29.6%) [3.67]	65 (22.7%) [2.70]	0.080	129
Cardiovascular mortality	54 (25.0%) [3.09]	53 (18.5%) [2.20]	0.094	107
Non-fatal myocardial infarction	27 (12.5%) [1.69]	16 (5.6%) [0.68]	0.006 *	43
Stroke/TIA	11 (5.1%) [0.66]	6 (2.1%) [0.25]	0.066	17
New PCI/CABG	44 (20.4%) [3.09]	31 (10.8%) [1.42]	0.003 *	75
Target vessel revascularization	27 (12.5%) [1.66]	14 (4.9%) [0.59]	0.002 *	41
Hospitalization for heart failure	36 (16.7%) [2.22]	17 (5.9%) [0.71]	<0.001 *	53

* *p* < 0.05 (statistically significant). Cell format: number of events (cumulative percentage of group) [incidence rate per 100 person-years]. *p*-values from χ^2^ test for the cumulative event proportions. Person-years computed as the sum of individual follow-up times until first event or administrative censoring at month 120. Total person-years: 1715.8 (MetS-positive) and 2411.6 (MetS-negative) for mortality; 1393.3 and 2155.8 for MACE; and 1625.2 and 2399.3 for heart failure. Abbreviations: TIA—transient ischemic attack; PCI—percutaneous coronary intervention; CABG—coronary artery bypass graft; PY—person-years.

## Data Availability

The data that support the findings of this study are available on request from the corresponding author.

## Supplementary Materials

The following supporting information can be downloaded at: https://www.mdpi.com/article/10.3390/medsci14020268/s1, Figure S1. Patient flow through the study (CONSORT-style flow diagram). Of 531 STEMI patients enrolled at the University Clinical Centre of Serbia between December 2009 and June 2010, 24 patients were lost to follow-up (alive but unreachable by telephone or at registered home addresses), and one additional patient was excluded because the follow-up time could not be ascertained. The final analytical sample comprised 506 patients, who were stratified by AHA/NHLBI metabolic syndrome status (MetS-positive: n = 216, 42.7%; MetS-negative: n = 290, 57.3%). Ten-year primary outcomes are summarised in the bottom panels; Figure S2. Use of cardioprotective medications at the first follow-up control (month 1) and at the 60-month follow-up control. *p*-values shown above each pair of bars indicate between-group differences (Chi-square test). Abbreviations: MetS(+)—patients with metabolic syndrome; MetS(−)—patients without metabolic syndrome; RAAS—renin-angiotensin-aldosterone system; ACE-I—angiotensin-converting enzyme inhibitor; ARB—angiotensin receptor blocker; Figure S3. Forest plot of hazard ratios (HR) with 95% confidence intervals from multivariate Cox proportional hazards regression for all-cause mortality, MACE, and heart failure hospitalization. The MetS row is highlighted in gold. Red markers indicate statistically significant predictors (*p* < 0.05); gray markers indicate non-significant predictors. Abbreviations: LVEF—left ventricular ejection fraction; MI—myocardial infarction; MetS—metabolic syndrome; CAD—coronary artery disease; MACE—major adverse cardiovascular events. * Continuous variable; Figure S4. SYNTAX score analysis. (A) Mean SYNTAX score in MetS(+) versus MetS(−) patients (19.10 ± 6.76 vs. 17.34 ± 6.83, *p* = 0.006). (B) Ten-year event rates for all-cause mortality, MACE, and heart failure hospitalization stratified by SYNTAX score tertiles: low (≤14, n = 155), mid (15–22, n = 166), and high (≥23, n = 142). (C) Spearman correlation between SYNTAX score and ten-year MACE rate (ρ = 0.337, *p* < 0.001). Abbreviations: MetS(+)—patients with metabolic syndrome; MetS(−)—patients without metabolic syndrome; MACE—major adverse cardiovascular events; SYNTAX—Synergy Between Percutaneous Coronary Intervention with Taxus and Cardiac Surgery; Figure S5. Obesity paradox analysis. (A) Kaplan-Meier cumulative mortality curves in non-obese patients stratified by MetS status (MetS(+) n = 35 vs. MetS(−) n = 109; log-rank *p* = 0.008). (B) Kaplan-Meier cumulative mortality curves in obese patients stratified by MetS status (MetS(+) n = 181 vs. MetS(−) n = 181; log-rank *p* = 0.529). (C) Excess ten-year mortality attributable to MetS in non-obese versus obese patients: 21.8 percentage points in non-obese patients versus 3.3 percentage points in obese patients. Obesity was defined as abdominal obesity using waist circumference thresholds (≥102 cm in men, ≥88 cm in women) consistent with AHA/NHLBI criteria. Abbreviations: MetS(+)—patients with metabolic syndrome; MetS(−)—patients without metabolic syndrome; Figure S6. MetS criteria burden and new-onset diabetes. (A) Ten-year event rates for all-cause mortality, MACE, and heart failure hospitalization stratified by the number of fulfilled MetS criteria (0–5). For heart failure, rates ranged from 8.3% at 0 criteria to 36.8% at 5 criteria. (B) Dose-response analysis restricted to MetS(+) patients (3–5 criteria): Spearman correlation between number of criteria and heart failure (ρ = 0.106, *p* = 0.123); no dose-response gradient was observed for MACE or mortality (ρ = −0.01 for both, *p* > 0.8). (C) New-onset type 2 diabetes mellitus during ten-year follow-up: 7.9% in MetS(+) versus 2.8% in MetS(−) patients (Chi-square *p* = 0.016). Abbreviations: MetS(+)—patients with metabolic syndrome; MetS(−)—patients without metabolic syndrome; MACE—major adverse cardiovascular events; DM—diabetes mellitus; Figure S7. Receiver-operating-characteristic (ROC) curves for the four machine-learning models across the three primary ten-year endpoints. (A) MACE; (B) all-cause mortality; (C) heart-failure hospitalization. Curves were generated from out-of-fold predicted probabilities in 10-fold stratified cross-validation (random_state = 42 throughout). Cross-validated areas under the curve (AUC) are shown in each panel’s legend, sorted in ascending order, with the highest-AUC model drawn on top. The dashed gray diagonal indicates chance classification. For all-cause mortality, all four models achieve comparable discrimination (AUC 0.825–0.834). For MACE, AUC values cluster around 0.72–0.75. For heart failure, logistic regression performs best (AUC 0.793), followed by random forest (0.775). LR, logistic regression (L2 regularization, C = 0.1); RF, random forest (300 trees, max depth 5); GBM, gradient-boosting machine (200 estimators, max depth 3, learning rate 0.05); XGBoost-equivalent, gradient boosting with 500 estimators, max depth 4, learning rate 0.03, row and column subsampling 0.8; Figure S8. Calibration curves for the best-performing model per endpoint, derived from out-of-fold predicted probabilities from 10-fold stratified cross-validation. (A) MACE—random forest; (B) all-cause mortality—gradient boosting; (C) heart-failure hospitalization—logistic regression. Predicted probabilities were sorted into eight quantile-based bins; observed event rates within each bin are plotted against mean predicted probabilities. Error bars are 95% binomial standard errors of the within-bin observed event rates. Bin sample sizes are annotated next to each point. The dashed gray diagonal indicates perfect calibration. Brier scores and AUC values for each panel are shown in the upper-left text box. The mortality model shows near-ideal calibration across the full range of predicted probabilities; MACE and heart-failure models show acceptable calibration, with minor systematic deviations in the lower- and middle-probability bins, consistent with smaller per-bin sample sizes for these endpoints. AUC, area under the ROC curve; Table S1. Cross-validated discrimination and calibration of the four machine-learning models for the three primary ten-year endpoints. Discrimination is reported as the area under the receiver operating characteristic curve (AUC), and calibration as the Brier score (mean squared error of predicted probabilities; lower values indicate better calibration). All values were computed from out-of-fold predicted probabilities using 10-fold stratified cross-validation with random_state = 42. The best-performing model per endpoint, selected by AUC, is highlighted in pale yellow and marked in the final block; this is the model used to generate the calibration curves shown in Supplementary Figure S8 and the risk-quartile analysis shown in Supplementary Figure S9; Figure S9. Observed ten-year event rates across quartiles of predicted probability, using the best-performing model per endpoint. (A) MACE—random forest; (B) all-cause mortality—gradient boosting; (C) heart-failure hospitalization—logistic regression. Patients were sorted by out-of-fold predicted probability and divided into four equal-sized quartiles (Q1 = lowest, Q4 = highest predicted risk; n ≈ 126–127 per quartile). The vertical axis shows the observed event rate within each quartile, with 95% binomial confidence intervals (Clopper–Pearson method).
