# Supplementary material for: Impact of MetS on Long-Term Prognosis Among STEMI Patients Treated with pPCI—Ten-Year Follow-Up Study
Source: Med Sci (Basel). 2026 May 21;14(2):268. doi: 10.3390/medsci14020268 (PMC13214653; doi:10.3390/medsci14020268)
Supplement: Supplementary file 1 [file medsci-14-00268-s001.zip › Supplementary_Material R2.pdf]

Supplementary Material

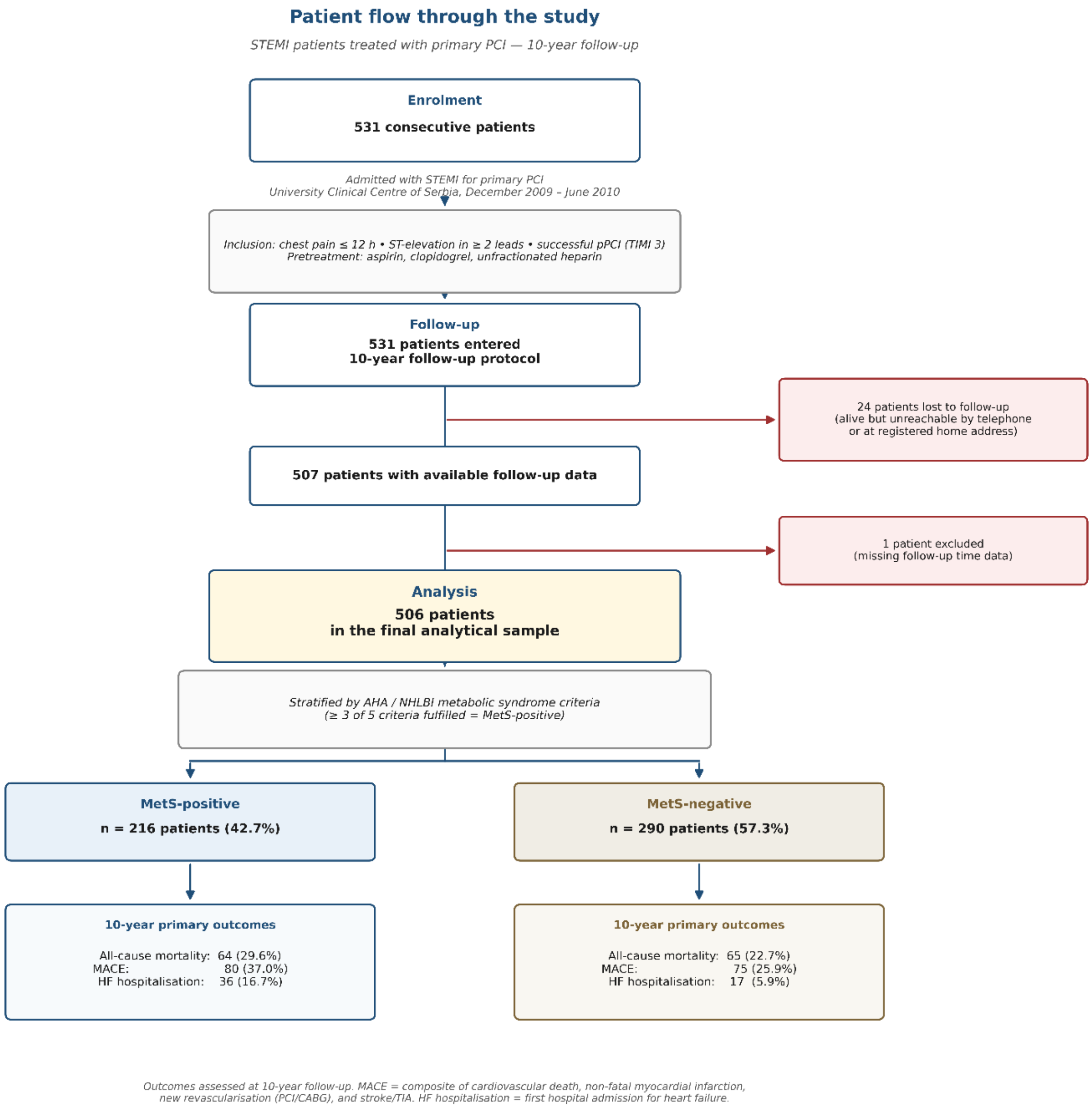

Figure S1. Patient flow through the study (CONSORT-style flow diagram). Of 531 STEMI patients enrolled at the University Clinical Centre of Serbia between December 2009 and June 2010, 24 patients were lost to follow-up (alive but unreachable by telephone or at registered home addresses), and one additional patient was excluded because the follow-up time could not be ascertained. The final analytical sample comprised 506 patients, who were stratified by AHA/NHLBI metabolic syndrome status (MetS-positive: n = 216, 42.7%; MetS-negative: n = 290, 57.3%). Ten-year primary outcomes are summarised in the bottom panels.

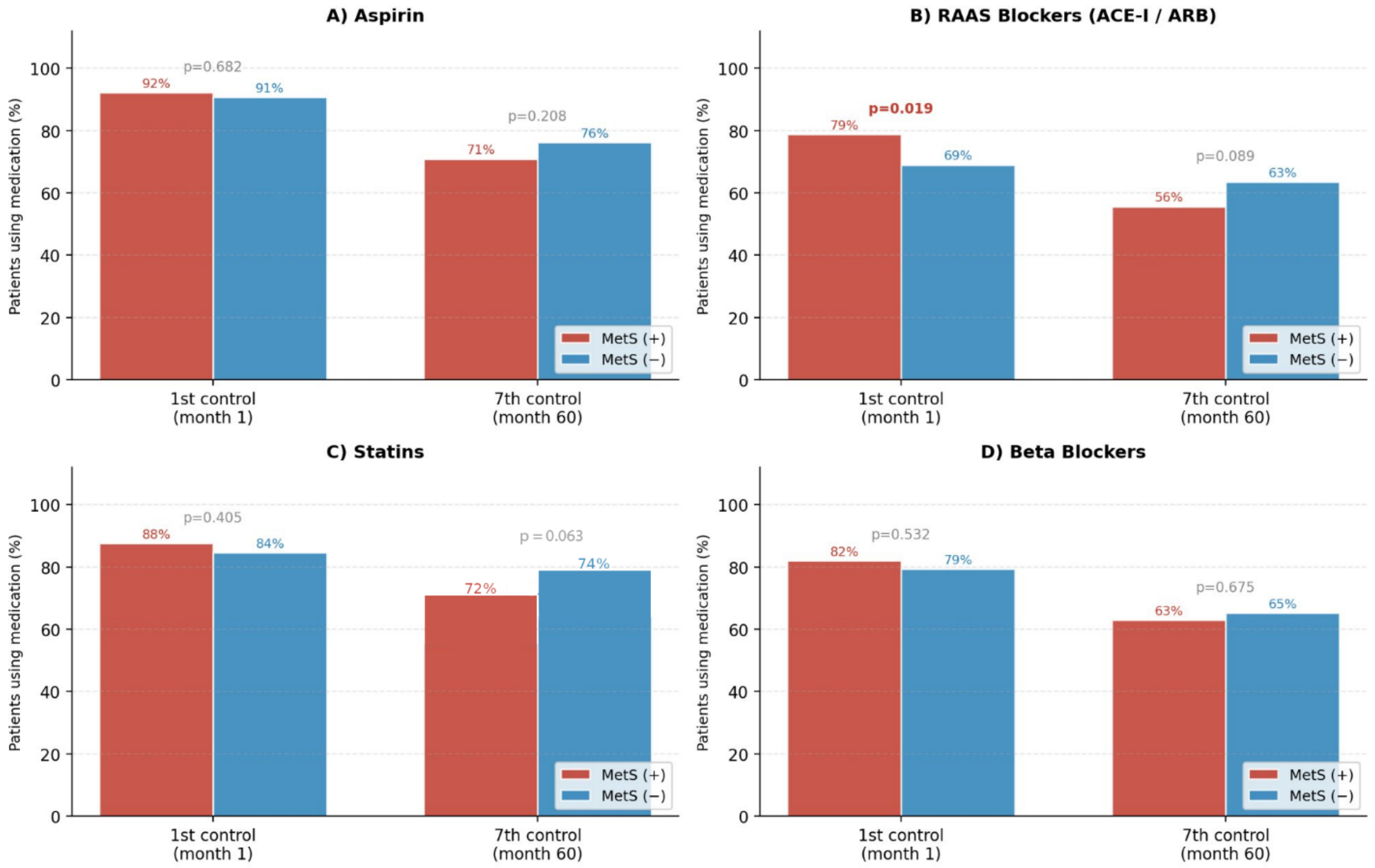

Figure S2. Use of cardioprotective medications at the first follow-up control (month 1) and at the 60-month follow-up control. p-values shown above each pair of bars indicate between-group differences (Chi-square test). Abbreviations: MetS(+) – patients with metabolic syndrome; MetS(−) – patients without metabolic syndrome; RAAS – renin-angiotensin-aldosterone system; ACE-I – angiotensin-converting enzyme inhibitor; ARB – angiotensin receptor blocker.

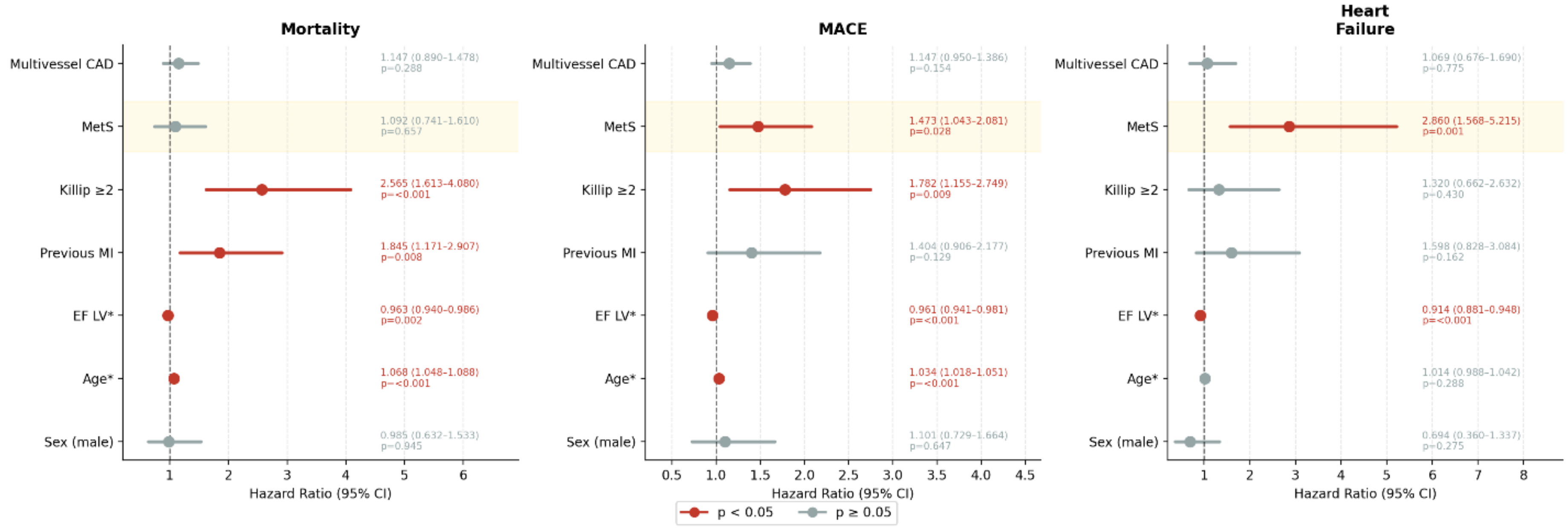

Figure S3. Forest plot of hazard ratios (HR) with 95% confidence intervals from multivariate Cox proportional hazards regression for all-cause mortality, MACE, and heart failure hospitalization. The MetS row is highlighted in gold. Red markers indicate statistically significant predictors ($p<0.05$); gray markers indicate non-significant predictors. Abbreviations: LVEF – left ventricular ejection fraction; MI – myocardial infarction; MetS – metabolic syndrome; CAD – coronary artery disease; MACE – major adverse cardiovascular events. * Continuous variable.

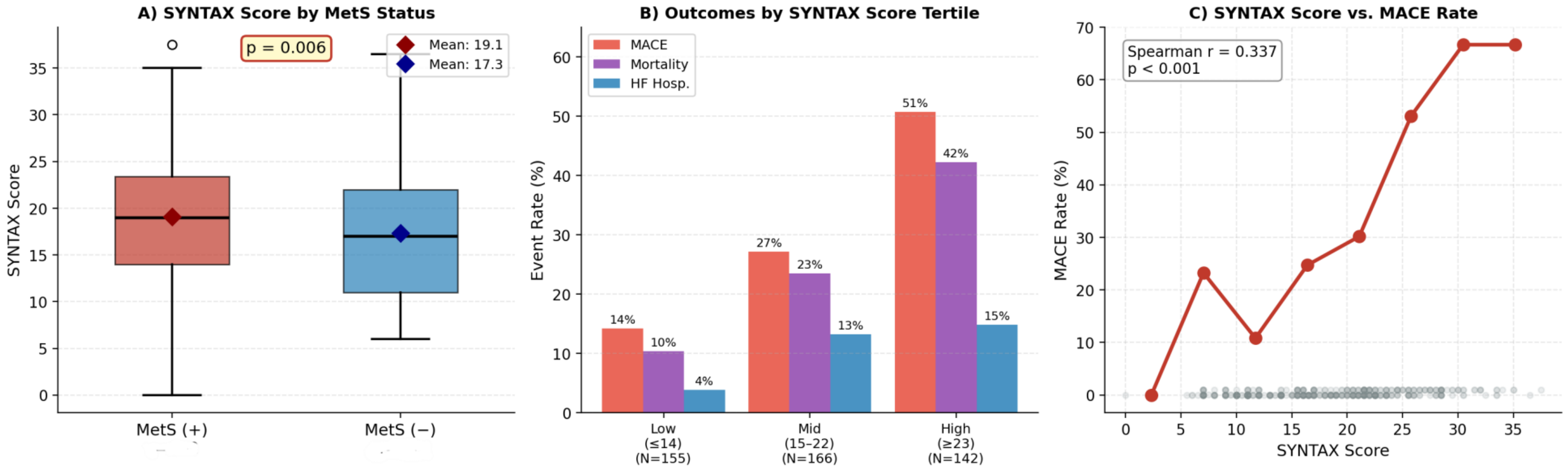

Figure S4. SYNTAX score analysis. (A) Mean SYNTAX score in MetS(+) versus MetS(−) patients (19.10±6.76 vs. 17.34±6.83, p=0.006). (B) Ten-year event rates for all-cause mortality, MACE, and heart failure hospitalization stratified by SYNTAX score tertiles: low (≤14, n=155), mid (15–22, n=166), and high (≥23, n=142). (C) Spearman correlation between SYNTAX score and ten-year MACE rate (ρ=0.337, p<0.001). Abbreviations: MetS(+) – patients with metabolic syndrome; MetS(−) – patients without metabolic syndrome; MACE – major adverse cardiovascular events; SYNTAX – Synergy Between Percutaneous Coronary Intervention with Taxus and Cardiac Surgery.

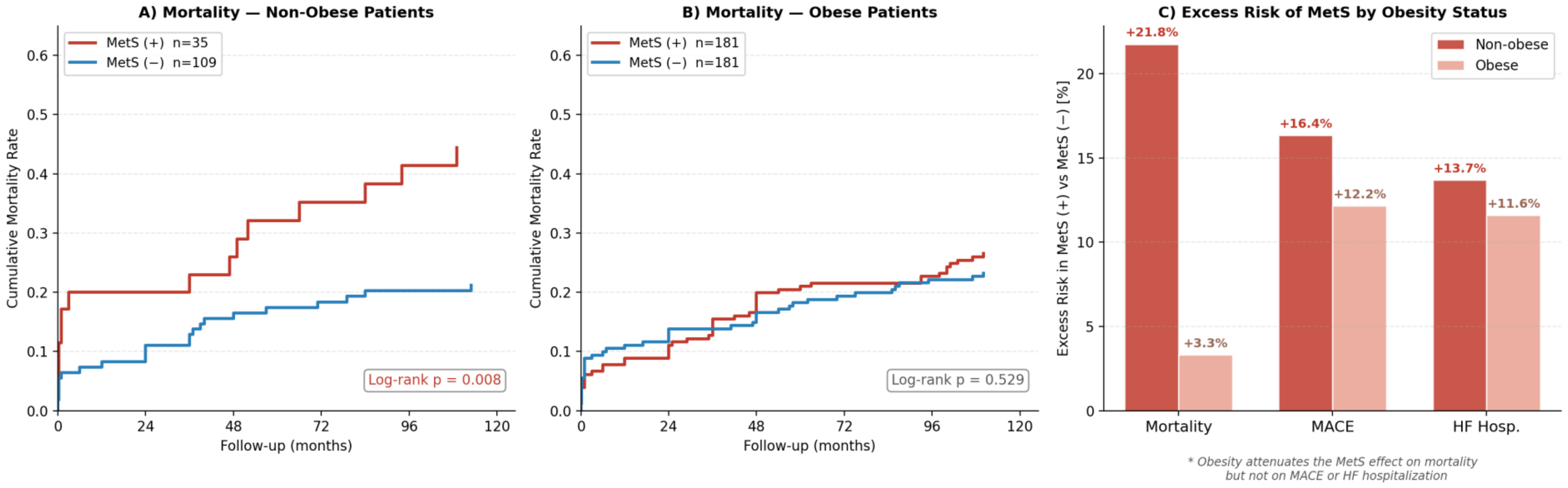

Figure S5. Obesity paradox analysis. (A) Kaplan-Meier cumulative mortality curves in non-obese patients stratified by MetS status (MetS(+) n=35 vs. MetS(−) n=109; log-rank p=0.008). (B) Kaplan-Meier cumulative mortality curves in obese patients stratified by MetS status (MetS(+) n=181 vs. MetS(−) n=181; log-rank p=0.529). (C) Excess ten-year mortality attributable to MetS in non-obese versus obese patients: 21.8 percentage points in non-obese patients versus 3.3 percentage points in obese patients. Obesity was defined as abdominal obesity using waist circumference thresholds (≥102 cm in men, ≥88 cm in women) consistent with AHA/NHLBI criteria. Abbreviations: MetS(+) – patients with metabolic syndrome; MetS(−) – patients without metabolic syndrome.

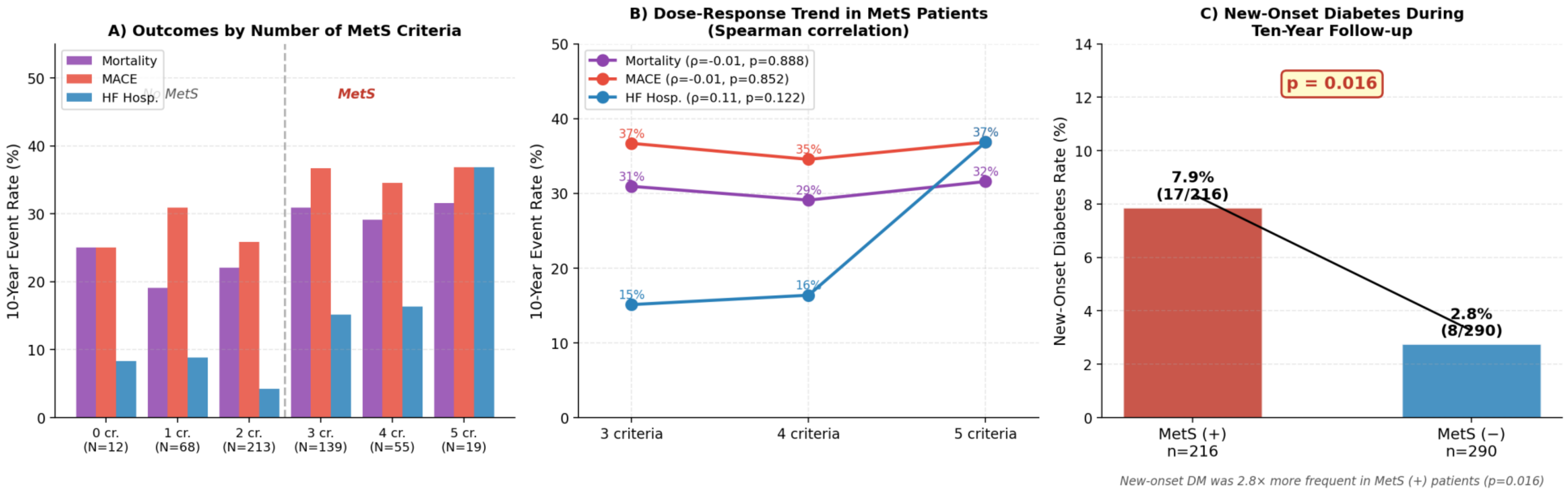

Figure S6. MetS criteria burden and new-onset diabetes. (A) Ten-year event rates for all-cause mortality, MACE, and heart failure hospitalization stratified by the number of fulfilled MetS criteria (0–5). For heart failure, rates ranged from 8.3% at 0 criteria to 36.8% at 5 criteria. (B) Dose-response analysis restricted to MetS(+) patients (3–5 criteria): Spearman correlation between number of criteria and heart failure (ρ=0.106, p=0.123); no dose-response gradient was observed for MACE or mortality (ρ=−0.01 for both, p>0.8). (C) New-onset type 2 diabetes mellitus during ten-year follow-up: 7.9% in MetS(+) versus 2.8% in MetS(−) patients (Chi-square p=0.016). Abbreviations: MetS(+) – patients with metabolic syndrome; MetS(−) – patients without metabolic syndrome; MACE – major adverse cardiovascular events; DM – diabetes mellitus.

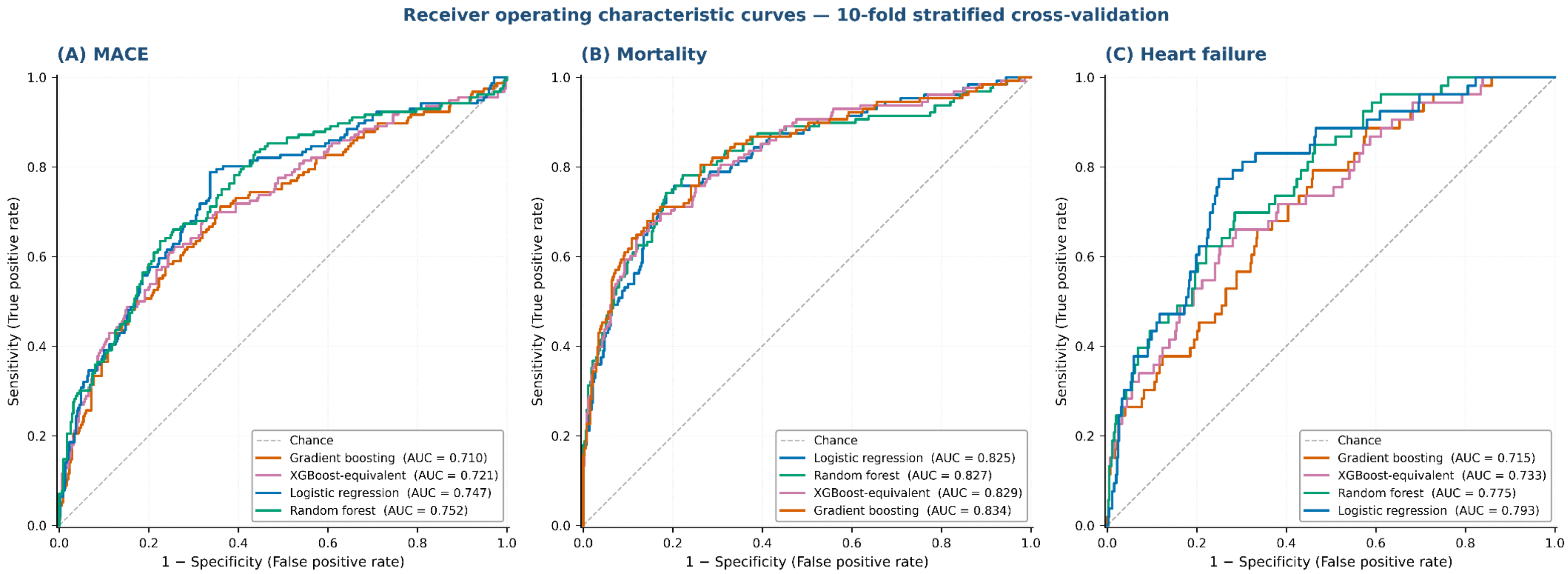

Figure S7. Receiver-operating-characteristic (ROC) curves for the four machine-learning models across the three primary ten-year endpoints. (A) MACE; (B) all-cause mortality; (C) heart-failure hospitalization. Curves were generated from out-of-fold predicted probabilities in 10-fold stratified cross-validation (random_state = 42 throughout). Cross-validated areas under the curve (AUC) are shown in each panel's legend, sorted in ascending order, with the highest-AUC model drawn on top. The dashed gray diagonal indicates chance classification. For all-cause mortality, all four models achieve comparable discrimination (AUC 0.825–0.834). For MACE, AUC values cluster around 0.72–0.75. For heart failure, logistic regression performs best (AUC 0.793), followed by random forest (0.775). LR, logistic regression (L2 regularization, C = 0.1); RF, random forest (300 trees, max depth 5); GBM, gradient-boosting machine (200 estimators, max depth 3, learning rate 0.05); XGBoost-equivalent, gradient boosting with 500 estimators, max depth 4, learning rate 0.03, row and column subsampling 0.8.

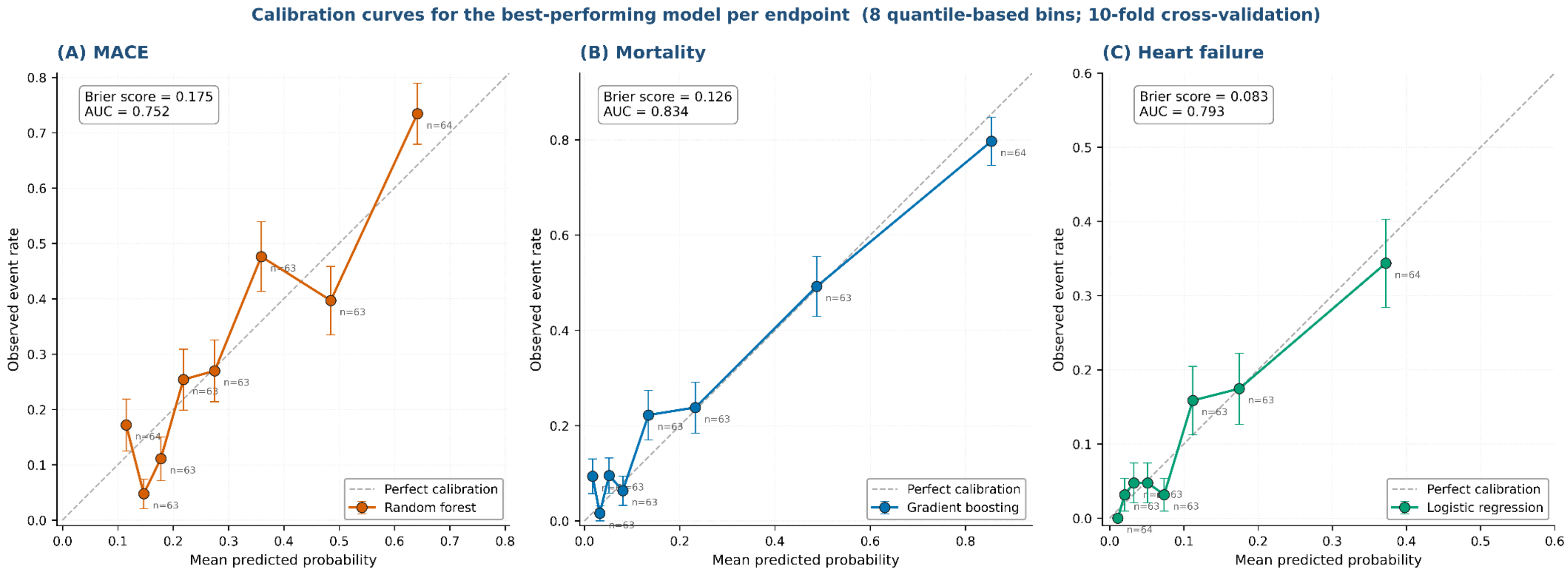

Figure S8. Calibration curves for the best-performing model per endpoint, derived from out-of-fold predicted probabilities from 10-fold stratified cross-validation. (A) MACE — random forest; (B) all-cause mortality — gradient boosting; (C) heart-failure hospitalization — logistic regression. Predicted probabilities were sorted into eight quantile-based bins; observed event rates within each bin are plotted against mean predicted probabilities. Error bars are 95% binomial standard errors of the within-bin observed event rates. Bin sample sizes are annotated next to each point. The dashed gray diagonal indicates perfect calibration. Brier scores and AUC values for each panel are shown in the upper-left text box. The mortality model shows near-ideal calibration across the full range of predicted probabilities; MACE and heart-failure models show acceptable calibration, with minor systematic deviations in the lower- and middle-probability bins, consistent with smaller per-bin sample sizes for these endpoints. AUC, area under the ROC curve.

Table S1. Cross-validated discrimination and calibration of the four machine-learning models for the three primary ten-year endpoints. Discrimination is reported as the area under the receiver operating characteristic curve (AUC), and calibration as the Brier score (mean squared error of predicted probabilities; lower values indicate better calibration). All values were computed from out-of-fold predicted probabilities using 10-fold stratified cross-validation with random_state = 42. The best-performing model per endpoint, selected by AUC, is highlighted in pale yellow and marked in the final block; this is the model used to generate the calibration curves shown in Supplementary Figure S8 and the risk-quartile analysis shown in Supplementary Figure S9.

Endpoint	Logistic regression	Random forest	Gradient boosting	XGBoost-equivalent
Discrimination — Area under the ROC curve (AUC; higher is better)				
**MACE**	0.747	**0.752**	0.710	0.721
**Mortality**	0.825	0.827	**0.834**	0.829
**Heart failure**	**0.793**	0.775	0.715	0.733
Calibration — Brier score (lower is better)				
**MACE**	0.176	**0.175**	0.191	0.196
**Mortality**	0.131	0.130	**0.126**	0.136
**Heart failure**	**0.083**	0.082	0.090	0.090
Best-performing model per endpoint (selected by AUC)				
**MACE**	—	✓ **Selected**	—	—
**Mortality**	—	—	✓ **Selected**	—
**Heart failure**	✓ **Selected**	—	—	—

AUC, area under the receiver-operating-characteristic curve; LR, logistic regression (L2 regularisation, C = 0.1); RF, random forest (300 trees, max depth 5); GB, gradient boosting (200 estimators, max depth 3, learning rate 0.05); XGBoost-equivalent, gradient boosting with 500 estimators, max depth 4, learning rate 0.03 and row/column subsampling 0.8; MACE, major adverse cardiovascular events.

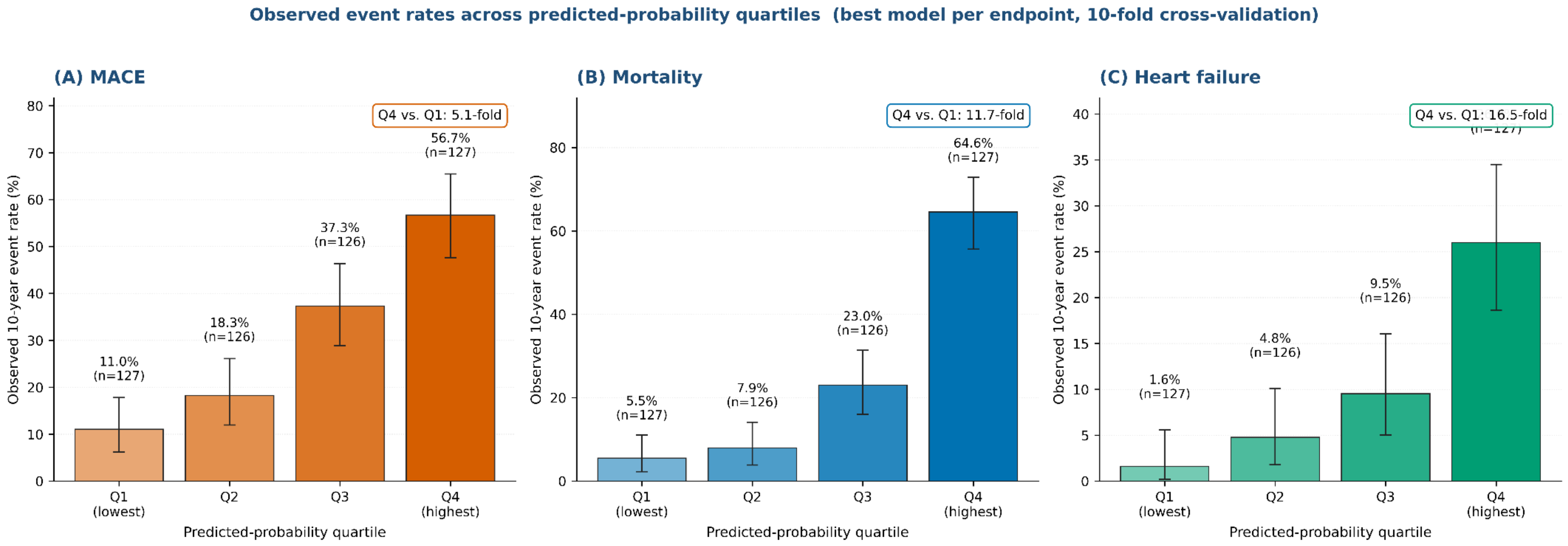

Figure S9. Observed ten-year event rates across quartiles of predicted probability, using the best-performing model per endpoint. (A) MACE — random forest; (B) all-cause mortality — gradient boosting; (C) heart-failure hospitalization — logistic regression. Patients were sorted by out-of-fold predicted probability and divided into four equal-sized quartiles (Q1 = lowest, Q4 = highest predicted risk; n ≈ 126–127 per quartile). The vertical axis shows the observed event rate within each quartile, with 95% binomial confidence intervals (Clopper–Pearson method).
